# Cost-effectiveness of screening for ovarian cancer amongst postmenopausal women: a model-based economic evaluation

**DOI:** 10.1186/s12916-016-0743-y

**Published:** 2016-12-06

**Authors:** Ben Kearns, Jim Chilcott, Sophie Whyte, Louise Preston, Susi Sadler

**Affiliations:** 1 The University of Sheffield, Regent Court, 30 Regent Street, Sheffield, S1 4DA UK; 2 University of Exeter Medical School, St Luke’s Campus, Heavitree Road, Exeter, EX1 2LU UK

**Keywords:** Ovarian neoplasm, Mass screening, Early detection of cancer, Health economics

## Abstract

**Background:**

The United Kingdom Collaborative Trial of Ovarian Cancer Screening (UKCTOCS) was the biggest ovarian cancer screening trial to date. A non-significant effect of screening on ovarian cancer was reported, but the authors noted a potential delayed effect of screening, and suggested the need for four years further follow-up. There are no UK-based cost-effectiveness analyses of ovarian cancer screening. Hence we assessed the lifetime outcomes associated with, and the cost-effectiveness of, screening for ovarian cancer in the UK, along with the value of further research.

**Methods:**

We performed a model-based economic evaluation. Effectiveness data were taken from UKCTOCS, which considered strategies of multimodal screening (MMS), ultrasound screening (USS) and no screening. We conducted systematic reviews to identify the remaining model inputs, and performed a rigorous and transparent prospective evaluation of different methods for extrapolating the effect of screening on ovarian cancer mortality. We considered costs to the UK healthcare system and measured effectiveness using quality-adjusted life years (QALYs). We used value of information methods to estimate the value of further research.

**Results:**

Over a lifetime, MMS and USS were estimated to be both more expensive and more effective than no screening. USS was dominated by MMS, being both more expensive and less effective. Compared with no screening, MMS cost on average £419 more (95% confidence interval £255 to £578), and generated 0.047 more QALYs (0.002 to 0.088). The incremental cost-effectiveness ratio (ICER) comparing MMS with no screening was £8864 per QALY (£2600 to £51,576). Alternative extrapolation methods increased the ICER, with the highest value being £36,769 (£13,888 to dominated by no screening). Using the UKCTOCS trial horizon, both MMS and USS were dominated by no screening, as they produced fewer QALYs at a greater cost. The value of research into eliminating all uncertainty in long-term effectiveness was estimated to be worth up to £20 million, or approximately £5 million for four years follow-up.

**Conclusions:**

Screening for ovarian cancer with MMS is both more effective and more expensive than not screening. Compared to national willingness to pay thresholds, lifetime cost-effectiveness is promising, but there remains considerable uncertainty regarding extrapolated long-term effectiveness.

**Electronic supplementary material:**

The online version of this article (doi:10.1186/s12916-016-0743-y) contains supplementary material, which is available to authorized users.

## Background

Ovarian cancer is the fifth highest cause of cancer deaths in women in the UK [1]. Survival is strongly associated with the stage of cancer at diagnosis, with women presenting with the most advanced stage of disease having one-year survival rates of 53% compared to 97% for women presenting with the least advanced stage of disease [2]. The majority of women with ovarian cancer present symptomatically with advanced disease [3]. Hence it has been hypothesised that screening has the potential to detect ovarian cancers earlier, which may in turn lead to improvements in survival.

The United Kingdom Collaborative Trial of Ovarian Cancer Screening (UKCTOCS) is the largest ovarian cancer screening trial to date [4]. It evaluated the performance of two screening strategies compared to no screening. The ultrasound screening (USS) strategy involved first line screening with a transvaginal ultrasound scan (TVS), carried out by certified sonographers or National Health Service (NHS) staff with similar experience (type 1). Second line screening involved a TVS performed by more experienced staff (type 2), including senior sonographers, radiologists or experienced gynaecologists. The multimodal screening (MMS) strategy involved first line screening with the CA-125 blood test interpreted using a risk of ovarian cancer algorithm (ROCA) [5] and second line screening with a type 2 TVS. Between April 2001 and October 2005, 202,638 women were randomised to one of the three trial arms: 101,359 to no screening, 50,639 to USS and 50,640 to MMS. Screening was performed until 31 December 2011, with a median of 11.1 years follow-up. The primary outcome was ovarian cancer mortality, with results showing that MMS was associated with a 15% relative reduction (95% confidence interval –3% to 30%, *p* = 0.10) [6]. A relative reduction of 11% was observed for USS (–7% to 27%, *p* = 0.21) [6]. Whilst neither of the mortality reductions reached conventional levels of statistical significance, the study authors noted a potential delayed effect of screening, and called for four additional years of follow-up to fully assess the extent of the mortality reductions [7].

Our model-based economic evaluation had two aims. The first was to evaluate the potential cost-effectiveness of screening for ovarian cancer in the UK. The second was to estimate the value of further research into ovarian cancer screening, including the value of reducing uncertainty in the long-term effect of screening on mortality.

## Methods

### Estimates of clinical effectiveness

We conducted a systematic review to identify any relevant screening trials in addition to the UKCTOCS. This identified an existing systematic review and meta-analysis by Reade et al. [8]. We did not identify any additional trials beyond those identified by Reade et al. [8], although two additional UKCTOCS publications were available [6, 9]. The UKCTOCS trial results are likely to supersede those of previous screening studies due to evolutions in the use of blood tests for screening. Hence effectiveness data were taken from the UKCTOCS study alone.

We used results in the public domain to estimate ovarian cancer incidence and mortality for all three trial arms. We digitised Kaplan-Meier curves [6] using EnGauge software and replicated individual patient data in R using the method described by Guyot et al. [10]. This replicated data covered the time horizon of the UKCTOCS trial, with a median follow-up of 11.1 years, and was validated by comparing model estimates of cancers diagnosed and ovarian cancer mortality at 11 years with the trial estimates. For the economic evaluation, estimates of lifetime clinical effectiveness were required. Hence we carried out a prospective evaluation of different methods for extrapolating the UKCTOCS trial data. The UKCTOCS triallists analysed the effects of screening on mortality as time-varying hazard ratios using Royston-Parmar (R-P) models [11]. For consistency, the base-case extrapolation of mortality data used R-P models to estimate the hazard of mortality for no screening, and hazard ratios for MMS and USS (both relative to no screening). Hazards and hazard ratios were extrapolated using time-series (exponential smoothing) methods [12].

The use of standard parametric models was also assessed in two structural uncertainty analyses: one using separate standard parametric models for each trial arm, and one using the same parametric model for all three arms. Five parametric models were considered (exponential, Weibull, Gompertz, log-logistic and log-normal), and the choice between the models was based on minimising the Bayesian information criterion, which was either calculated separately for each trial arm or combined across the three arms. We used a novel model discrepancy method to explore the potential impact of structural uncertainty associated with the extrapolation of long-term outcomes in an additional structural uncertainty analysis [13]. This had the dual effect of reducing the effectiveness of both MMS and USS during the extrapolated period (as compared with the base-case estimates) and also increasing the uncertainty in these estimates. The model discrepancy tested was a cumulative 5% decrease in the effect of screening per year, with a standard deviation of 0.05. Incidence data were extrapolated using standard parametric survival models. Estimates of the number of false positives (per cancer diagnosed) were taken from the UKCTOCS [6].

Other-cause mortality was taken from the UKCTOCS publication (Appendix Web Table 6 of Jacobs et al 2016 [6]), which presented the number of deaths and women-years by screening arm. As there was no evidence that other-cause mortality varied with screening arm, this evidence was pooled to obtain an overall annual value of 0.61% (13,296 deaths from 2,194,447 women-years). Other-cause mortality was extrapolated using National Life Tables [14], and assuming that women would be on average 60 when they enter the model (so that after the within-trial period has ended, women would be on average 72 years old). This approach to separately modelling mortality due to ovarian cancer and mortality due to other causes is consistent with existing economic evaluations of ovarian cancer [15, 16].

### Utility data

We performed a systematic review which identified two published systematic reviews [8, 17] and two studies which provided ovarian cancer utility values [9, 18]. The systematic review of Hess et al. [17] focussed on the treatment of ovarian cancer. There was a lack of consistent evidence on the effect of different treatment strategies on women’s health-related quality of life (HRQoL), which may be due to the heterogeneity in the utility measures used, treatments received and populations considered. However, there was a statistically significant increase in HRQoL after completing treatment, compared with at the start of treatment. The systematic review by Reade et al. was the previously discussed review of trials [8]. This identified three trials which evaluated the impact of screening on HRQoL. Reade et al. concluded that there was high-quality evidence to suggest that screening for ovarian cancer does not have an impact on HRQoL. This conclusion was supported by the two identified studies [9, 18]. Of these, the study by Havrilesky et al. also reported time trade-off valuations of utility for women diagnosed with early and advanced ovarian cancer [18]; these valuations were used to generate stage-specific disutility values for newly diagnosed ovarian cancer. We assumed that this disutility related to treatment, and only lasted for one year, after which women’s HRQoL returned to that of the general population. We further assumed that there was no disutility associated with receiving a screen, but that women receiving treatment following a false positive result would experience the same disutility as a woman with Stage 1 cancer.

### Costs and resource use

We derived costs and resource usage from sources including clinical guidelines, literature, data from the English Cancer Registries and estimates provided by multidisciplinary teams responsible for the management and treatment of ovarian cancer.

Resource use for ovarian cancer screening was taken from the UKCTOCS [4]. There are no routine estimates for either the cost of the CA-125 blood test or the ROCA algorithm. For the former we used cost estimates developed for the Early Cancer Detection Consortium [19]. As the ROCA was developed and trialled with support from public and charitable monies, we assumed that using the ROCA would not lead to any increase in the cost of a screen within the UK setting. The costs of type 1 and type 2 TVSs were derived from national reference costs.

Initial estimates for the cost of ovarian cancer diagnosis and treatment were taken from the Incisive report, which was commissioned by Cancer Research UK to examine the financial implications of achieving earlier diagnosis of colorectal, lung and ovarian cancers [20]. These estimates were supplemented by treatment data from the English Cancer registries and shared with two multidisciplinary teams (based in Sheffield and Birmingham), who refined the estimates of cost and resource use. These were used to derive estimates for the cost of diagnosing and treating ovarian cancer, based on stage at diagnosis.

An overview of the key model inputs is provided in Table 1. Further details on the derivation of utility and cost values, along with details of all the search strategies, are provided in Additional file 1.

**Table 1:** Key model inputs

	Mean	Distribution	95% confidence interval
Cost parameters			
Multimodal screening (drop-outs)	£54	Beta	£33 to £74
Multimodal screening (complete screening)	£61	Hybrid^a^	£37 to £83
Ultrasound screening (drop-outs)	£56	Gamma	£29 to £92
Ultrasound screening (complete screening)	£61	Hybrid^a^	£31 to £100
Screening invitation (either strategy)	£2.09	Gamma	£1.70 to £2.52
Diagnosis: Borderline	£110	Gamma	£91 to £135
Diagnosis: Stage 1 ovarian cancer	£116	Gamma	£90 to £133
Diagnosis: Stage 2 ovarian cancer	£126	Gamma	£94 to £140
Diagnosis: Stage 3 ovarian cancer	£126	Gamma	£102 to £152
Diagnosis: Stage 4 ovarian cancer	£112	Gamma	£102 to £152
Treatment: Borderline	£3000	Gamma	£1161 to £5696
Treatment: Stage 1 ovarian cancer	£6961	Gamma	£4856 to £9438
Treatment: Stage 2 ovarian cancer	£7325	Gamma	£5211 to £9795
Treatment: Stage 3 ovarian cancer	£9016	Gamma	£6866 to £11,454
Treatment: Stage 4 ovarian cancer	£5892	Gamma	£3823 to £8402
End of life cost: ovarian cancer	£7080	Gamma	£5761 to £8533
Utility parameters			
Utility cancer free	0. 900	Beta	0.325 to 1
Disutility Stage 1 ovarian cancer or false positive result	0.200	Beta	0.044 to 0.437
Disutility Stage 2 ovarian cancer	0.325	Beta	0.214 to 0.534
Disutility Stage 3 ovarian cancer	0.413	Beta	0.329 to 0.600
Disutility Stage 4 ovarian cancer	0. 455	Beta	0.413 to 0.601
Number of false positives per cancer identified			
Multimodal screening	2.302	Beta	2.188 to 2.412
Ultrasound screening	9.963	Beta	9.813 to 10.104

^a^Mixture of gamma and beta distributions; see Additional file 1 for more details

### Cost-effectiveness modelling methods

**Fig. 1:**
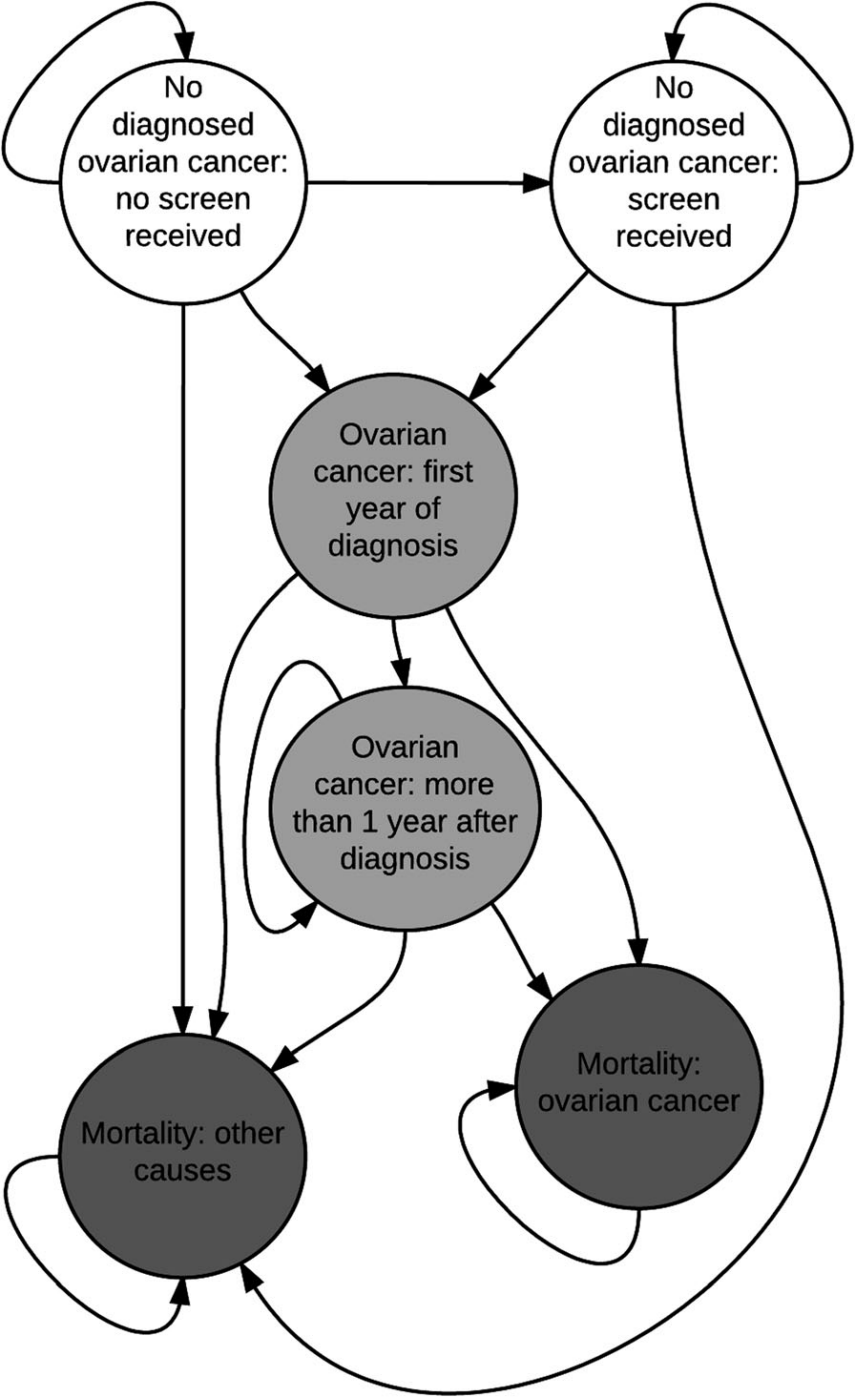
Schematic of the health economic model

The health economic model was a cohort-level Markov model developed in Microsoft Excel®, with the perspective of the NHS and Personal Social Services, a lifetime horizon and an annual cycle. Details on the derivation of annual transition probabilities from time-series and parametric survival models are provided in Additional file 1. Costs were reported in 2013/2014 pound sterling. The primary outcome was the incremental cost-effectiveness ratio (ICER) measured by the incremental cost per quality-adjusted life years (QALYs) gained. Both costs and QALYs were discounted at 3.5% per annum as recommended by the National Institute for Health and Care Excellence in England [21]. The model structure included six health states corresponding to the screening status of women without diagnosed ovarian cancer, diagnosed ovarian cancer stratified by the first and subsequent years of diagnosis, and mortality due to either ovarian cancer or other causes. The half-cycle correction was employed. A model schematic is provided in Fig. 1.

Estimates of cost-effectiveness were obtained by running the health economic model probabilistically over 5000 Monte Carlo samples, with percentile-based confidence intervals. In addition to the previously described structural uncertainty analyses, univariate sensitivity analyses were undertaken to examine the impact of individual model inputs on cost-effectiveness. A full description of these sensitivity analyses is provided in Additional file 1.

As part of the uncertainty analysis, value of information methods were used to estimate the value of further research. These methods assess the impact of uncertainty in the model results on decision uncertainty and the subsequent opportunity cost that is due to decision uncertainty. The population expected value of perfect information (EVPI) provides an estimate of how much should be spent to eliminate all decision uncertainty in the cost-effectiveness results. The expected value of perfect parameter information (EVPPI) estimates the contribution of each model input to the EVPI [22]. Estimates were obtained from the online Sheffield Accelerated Value of Information application (http://savi.shef.ac.uk/SAVI/), using standard methods for EVPI [23, 24] and EVPPI methods recently reported by Strong et al. [22]. It was assumed that the annual number of women affected by the decision about whether or not to fund screening was 7,121,000, equivalent to the Office for National Statistics Mid-2010 Population Estimates for women aged from 50 to –74 [25]. This value was adjusted by the uptake to UKCTOCS and subsequent compliance with screening [6] to give a value of 1,045,914. A time horizon for the decision of one year was used, as the population value implicitly covers all of the relevant years.

## Results

### Base-case cost-effectiveness results

The lifetime cost-effectiveness results for the three screening strategies are presented in Table 2. Compared to no screening, both active screening strategies were predicted to increase QALYs but also increased costs. The estimated total lifetime average cost per woman for no screening was £179 (95% confidence interval £137 to £242). Screening with MMS increased costs by £419 (£255 to £578), whilst for USS the increase was £646 (£386 to £973). The average QALYs accrued under no screening were 14.290 (5.159–15.907), with increases of 0.047 (0.002–0.088) and 0.007 (–0.042 to 0.049) for MMS and USS respectively. The ICER comparing MMS with no screening was £8864 per QALY (£2600 to £51,576). Use of USS was dominated by MMS, being both more expensive and less effective. Compared to a strategy of no screening, both MMS and USS resulted in increases in life expectancy of 0.58% and 0.28% respectively, equating to an extra 7.39 (1.58–12.65) and 3.58 (–3.46 to 8.76) weeks. With no screening, 3.19% (1.98–5.17%) of women would die from ovarian cancer. For MMS and USS, this proportion was estimated to reduce to 1.41% (0.56–3.46%) and 2.35% (1.06–5.21%) respectively. The estimated ICER for MMS (£8864) is lower than both the traditional willingness to pay levels for the NHS of £20,000 per QALY [26] and the recently suggested value of £13,000 per QALY [27]. For both MMS and USS, the cost of screening was approximately £400 per woman. Compared to a strategy of no screening, both active screening strategies resulted in increased treatment costs (due to the additional treatment of false positives) but lower end-of-life costs (due to fewer ovarian cancer deaths).

**Table 2:** Base-case probabilistic cost-effectiveness results: lifetime average costs and QALYs per woman

Model lifetime results	No screening	MMS	USS
Costs	£179	£598	£824
(95% confidence interval)	(£137 to £242)	(£434 to £758)	(£566 to £1154)
Incremental costs	–	£419	£646
(95% confidence interval)		(£255 to £578)	(£386 to £973)
QALYs	14.290	14.357	14.297
(95% confidence interval)	(5.159 to 15.907)	(5.168 to 15.959)	(5.147 to 15.926)
Incremental QALYs	–	0.047	0.007
(95% confidence interval)		(0.002 to 0.088)	(–0.042 to 0.049)
ICER per QALY	–	£8864 vs no screening	Dominated by MMS
(95% confidence interval)		(£2600 to £51,576)	
Life years	24.660	24.803	24.729
(95% confidence interval)	(24.543 to 24.741)	(24.668 to 24.872)	(24.554 to 24.822)
Incremental life years	–	0.142	0.069
(95% confidence interval)		(0.03 to 0.24)	(–0.07 to 0.17)
Ovarian cancer deaths	3.19%	1.41%	2.35%
(95% confidence interval)	(1.98% to 5.17%)	(0.56% to 3.46%)	(1.06% to 5.21%)
Incremental OC deaths	–	–1.77%	–0.83%
(95% confidence interval)		(–3.38% to –0.19%)	(–2.39% to 1.26%)
Model 11-year results	No screening	MMS	USS
Costs	£58	£510	£612
(95% confidence interval)	(£49 to £67)	(£350 to £660)	(£399 to £886)
QALYs	8.250	8.247	8.239
(95% confidence interval)	(2.823 to 9.163)	(2.820 to 9.162)	(2.801 to 9.157)
Life years	11.093	11.093	11.093
(95% confidence interval)	(11.090 to 11.096)	(11.086 to 11.098)	(11.089 to 11.097)
Cancers diagnosed	0.59%	0.65%	0.61%
(95% confidence interval)	(0.55% to 0.64%)	(0.58% to 0.72%)	(0.55% to 0.69%)
Ovarian cancer deaths	0.34%	0.31%	0.33%
(95% confidence interval)	(0.29% to 0.41%)	(0.21% to 0.44%)	(0.26% to 0.41%)
Observed trial results	No screening	MMS	USS
Cancers diagnosed	0.62%	0.67%	0.62%
(95% confidence interval)	(0.58% to 0.67%)	(0.60% to 0.74%)	(0.56% to 0.69%)
Ovarian cancer deaths	0.34%	0.29%	0.30%
(95% confidence interval)	(0.31% to 0.38%)	(0.25% to 0.34%)	(0.26% to 0.36%)

*OC* ovarian cancer, *QALYs* quality-adjusted life years

Costs and QALYs are discounted, life years are not. Incremental values are relative to no screening

A comparison of 11-year estimates with the UKCTOCS results (Table 2) shows close agreement for both the proportion of cancers diagnosed and the proportion of deaths due to ovarian cancer. At this time horizon, both MMS and USS were dominated by a strategy of no screening, being both more expensive and generating fewer QALYs, as the dis-benefits of screening — earlier treatment of asymptomatic women and treatment of false positives — outweighed the mortality reduction. Both MMS and USS remained dominated by no screening at 11 years in an exploratory analysis that removed the impact of false positives.

### Extrapolation assumptions

**Fig. 2:**
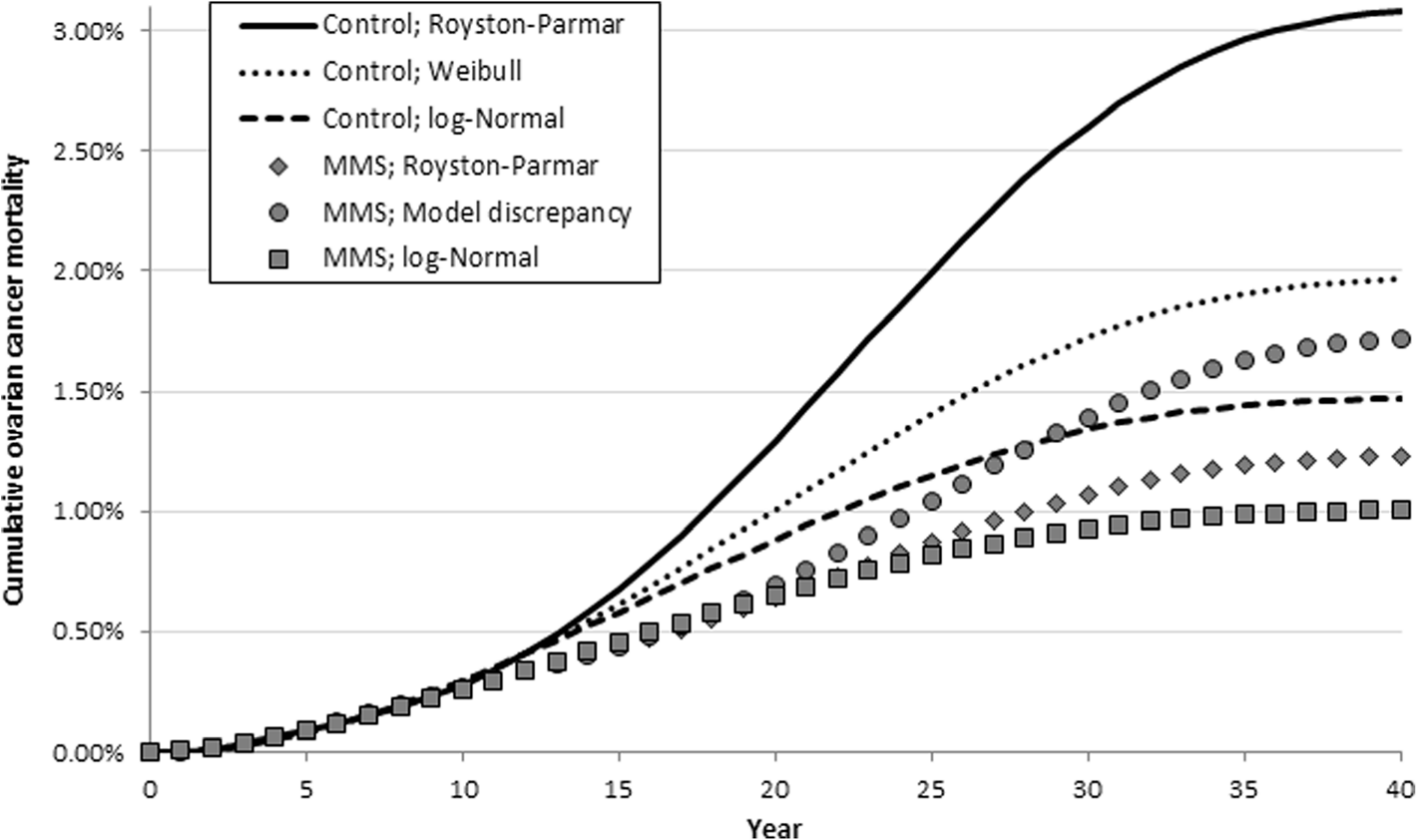
Cumulative ovarian cancer mortality for different extrapolation methods; MMS and no screening. *MMS* multimodal screening

When using separate parametric models for each trial arm, the log-normal was selected for both MMS and USS, whilst the Weibull was selected for no screening. When fitting the same model to all three trial arms, the log-normal was selected. Figure 2 displays estimates of cumulative ovarian cancer mortality for MMS and no screening for the extrapolation approaches considered, with cost-effectiveness results presented in Table 3. The use of parametric curves for extrapolation results in a lower estimated lifetime number of ovarian cancer deaths across all three trial arms, with the largest reductions observed for the no screening group (reduced from 3.19% to 1.99% or 1.49% for the Weibull and log-normal models respectively). This affects the ICER for MMS, which relative to no screening increases from £8864 in the base case to either £18,372 or £36,769 depending on the parametric assumptions employed. Introducing a model discrepancy increased the ICER for MMS relative to no screening to £12,643.

**Table 3:** Estimates of lifetime costs, effects and cost-effectiveness for different extrapolation assumptions

Separate parametric models	No screening	MMS	USS
Costs	£143	£588	£782
(95% confidence interval)	(£121 to £169)	(£425 to £745)	(£526 to £1110)
QALYs	14.352	14.376	14.361
(95% confidence interval)	(5.100 to 15.922)	(5.108 to 15.95)	(5.095 to 15.941)
ICER per QALY	–	£18,372 vs no screening	Dominated by MMS
(95% confidence interval)		(£7709 to £96,784)	
Life years	24.743	24.820	24.818
(95% confidence interval)	(24.706 to 24.771)	(24.789 to 24.840)	(24.786 to 24.839)
Ovarian cancer deaths	1.99%	1.04%	1.05%
(95% confidence interval)	(1.65% to 2.45%)	(0.85% to 1.34%)	(0.87% to 1.37%)
Same parametric models	No screening	MMS	USS
Costs	£128	£587	£780
(95% confidence interval)	(£109 to £149)	(£427 to £745)	(£520 to £1105)
QALYs	14.343	14.356	14.341
(95% confidence interval)	(5.174 to 15.933)	(5.176 to 15.949)	(5.166 to 15.94)
ICER per QALY	–	£36,769 vs no screening	Dominated by MMS
(95% confidence interval)		(£13,888 to dominated)	
Life years	24.778	24.821	24.818
(95% confidence interval)	(24.751 to 24.797)	(24.789 to 24.84)	(24.787 to 24.838)
Ovarian cancer deaths	1.49%	1.03%	.05%
(95% confidence interval)	(1.29% to 1.77%)	(0.85% to 1.35%)	(0.86% to 1.36%)
Model discrepancy; 5% per year	No screening	MMS	USS
Costs	£179	£614	£851
(95% confidence interval)	(£136 to £235)	(£440 to £784)	(£578 to £1194)
QALYs	14.209	14.244	14.194
(95% confidence interval)	(4.671 to 15.903)	(4.66 to 15.954)	(4.622 to 15.914)
ICER per QALY	–	£12,643 vs no screening	Dominated by MMS
(95% confidence interval)		(£3734 to dominated)	
Life years	24.659	24.800	24.722
(95% confidence interval)	(24.548 to 24.739)	(24.591 to 24.863)	(24.417 to 24.801)
Ovarian cancer deaths	3.22%	1.99%	3.42%
(95% confidence interval)	(2.02% to 5.08%)	(0.70% to 4.73%)	(1.37% to 7.59%)

*ICER* incremental cost-effectiveness ratio, *QALYs* quality-adjusted life years

### Estimates of uncertainty and the value of further research

**Fig. 3:**
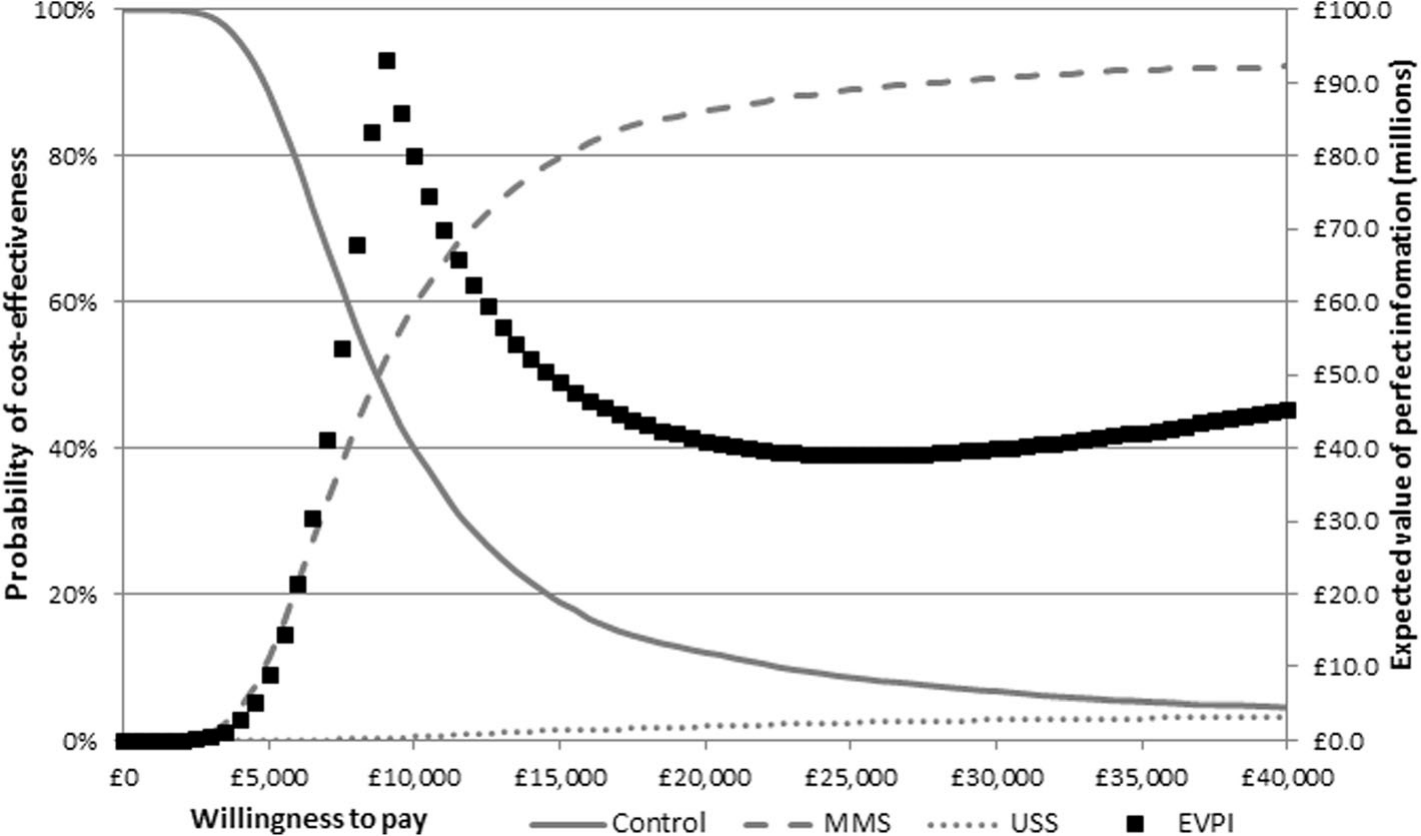
The probability that each screening strategy is cost-effective for different willingness to pay values (left axis) and the expected value of perfect information (right axis). *EVPI* expected value of perfect information, *MMS* multimodal screening, *USS* ultrasound screening

The probability of each of the screening strategies being cost-effective is displayed in Fig. 3 for maximum willingness to pay (WtP) values between £0 and £40,000 per QALY gained, with EVPI estimates on a separate axis. A WtP value indicates the monetary equivalent that is attributed to a QALY. For example, a willingness to pay of £20,000 indicates that a decision-maker is willing to pay £20,000 per QALY gained.

As WtP increases, more weight is given to effectiveness and the probability of MMS being cost-effective increases. At a WtP value of £13,000 the probability of MMS being cost-effective is 74.2%; at a value of £20,000 it is 86.2%. Population EVPI reaches a maximum at a WtP threshold of about £9000, with a value of approximately £90 million. Estimates of EVPPI, taken at a WtP of £20,000, show that the key drivers of decision uncertainty are long-term mortality effectiveness estimates for MMS compared to no screening, and the HRQoL for women with ovarian cancer. These contribute to 53% and 22% of the decision uncertainty respectively; it would be worth spending approximately £20 million to eliminate all of the uncertainty in the long-term effectiveness of screening.

Full details of the sensitivity analyses results are provided in Additional file 1. Of these analyses, cost-effectiveness results were sensitive to the inclusion of a disutility due to screening, the inclusion of a cost for the ROCA Test and the assumed cost of MMS. Including an annual disutility due to screening of 0.01 (equivalent to experiencing a disutility of 0.06 for a month) led to both screening strategies being dominated by no screening. With a disutility of 0.005 (equivalent to experiencing a disutility of 0.06 for a month) the ICER for MMS compared to no screening increased by more than 200% to £26,173 per QALY. Including a cost for the ROCA Test led to MMS being more expensive than USS. However, USS was extendedly dominated by the combination of no screening and MMS (ICER for USS vs no screening: £64,231 per QALY). The ICER for MMS compared to no screening increased by 260% to £30,552. Using the high cost estimate for MMS (with no ROCA cost) led to a relative increase in the ICER for MMS of 23%, whilst using the low estimate led to a relative reduction of 40%. Assuming that diagnosis in primary care has the same false-positive rate as MMS leads to a 21% relative reduction in the ICER for MMS. With the exception of the screening disutilities, for each of the sensitivity analyses considered, the corresponding ICER remained below the £13,000 WtP threshold.

## Discussion

Our two aims were to evaluate the lifetime cost-effectiveness of ovarian cancer screening and the value of further research. The results of our economic evaluation suggest that either an MMS or USS screening strategy is likely to result in health benefits when compared to no screening, but at increased costs. Screening using MMS is estimated to be both more effective and cheaper than USS. The base-case lifetime ICER comparing MMS against no screening was estimated to be £8864 per QALY (95% confidence interval £2600 to £51,576). Based on an 11-year time horizon, both MMS and USS are dominated by a strategy of no screening.

Value of information analyses suggested that at a willingness to pay of £20,000 per QALY it was worth spending approximately £20 million to eliminate all of this long-term uncertainty in the effect of screening on ovarian cancer mortality. The UKCTOCS triallists’ proposal to extend follow-up by four additional years is unlikely to resolve all uncertainty. In this model, the extrapolated period included 40.9 women-years across the three screening strategies, whilst the first four years of extrapolation provided 10.7 women-years. Hence, as a crude approximation, it may be worth spending £5.2 million to extend the UKCTOCS trial by four years. If willingness to pay values were nearer to the central estimate of cost-effectiveness (£8864 per QALY), as would happen if the threshold of £13,000 per QALY were used [27], then estimates of how much should be spent would increase.

The 2015 UKCTOCS publication, based on a median of 11.1 years follow-up, found that screening for ovarian cancer with MMS was associated with a non-significant ovarian cancer mortality reduction of 15%. The authors noted that this reduction could be decomposed into a non-significant reduction of 8% during the first seven years and a significant reduction of 23% in the last three years, representing a potential delayed effect of screening. Our within-trial analysis replicated the finding of no significant effect. When modelling lifetime outcomes, we extrapolated a time-varying late treatment effect. This led to MMS being associated with a significant reduction in ovarian cancer mortality over a lifetime. However, results were sensitive to the use of different extrapolation methods. There also remains the possibility that the observed mortality reduction may reflect a delayed time to ovarian cancer mortality [28]. If this is the case, then the mortality effect would vanish over a lifetime horizon. This extreme possibility is captured within the model discrepancy sensitivity analysis, for which there is no significant difference in ovarian cancer mortality between screening and not screening.

It is unreasonable to assume that the observed screening effect will vanish immediately at the 11-year time point; therefore, we used transparent methods of extrapolation that allowed us to explore the potential impact of alternative estimates of long-term effectiveness on decision uncertainty. The base-case analysis combined the flexibility of the R-P approach for within-trial estimates with a novel time-series method for extrapolation. A further innovation of this study was the incorporation of the model discrepancy method within the extrapolation to account for structural uncertainty associated with extrapolation. We are not aware of any other economic evaluations that have used this innovative combination.

In addition to the prospective evaluation of extrapolation methods, strengths of our study were the systematic reviews undertaken to inform the work and the timeliness of this work. All of the published economic evaluations of screening for ovarian cancer were conducted prior to the emergence of data from UKCTOCS on the effect of screening on mortality, and none used a UK healthcare perspective. In addition, ours is the first economic evaluation of screening for ovarian cancer to consider HRQoL as an outcome and so provide a more comprehensive assessment of the potential benefits and harms of screening for ovarian cancer amongst postmenopausal women. It is also the first study to estimate the value of further research into screening for ovarian cancer.

A limitation of our study was the lack of age and stage breakdowns for both the incidence of, and mortality from, ovarian cancer. This limited the analysis in that it was not possible to use the trial evidence to understand the natural history of ovarian cancer and thus to estimate the potential cost-effectiveness of alternative screening strategies, such as different screening intervals or different age ranges. The key remaining uncertainties in the health economics of ovarian cancer screening are the impact of the screening process on HRQoL, the cost of the ROCA Test and the long-term estimates of the effect of screening on ovarian cancer mortality. Different assumptions about how to model this led to twofold and fourfold increases in the ICER, from £8864 to £18,372 and £36,769.

## Conclusions

In conclusion, results from the UKCTOCS demonstrated that screening for ovarian cancer was associated with a non-statistically significant effect on ovarian cancer mortality, based on a median of 11 years of follow-up. The UKCTOCS triallists called for a further four years of follow-up to confirm or refute this finding. Based on 11 years of follow-up we estimated that screening is not cost-effective, as the mortality benefit is outweighed by the dis-benefits associated with both treating false positives and earlier treatment of women with ovarian cancer. In contrast, lifetime cost-effectiveness results are promising, with an estimated ICER comparing MMS with no screening of £8864 per QALY (95% confidence interval £2600 to £51,576). However, an evaluation of different extrapolation methods along with value of information methods showed that there is substantial uncertainty in the long-term effectiveness of MMS in reducing ovarian cancer mortality, which is a key driver of cost-effectiveness.

## Additional file

Additional file 1: Section A: Derivation of utility values for the health economic model. Section B: Estimates of resource use and costs for the health economic model. Section C: Derivation of annual transition probabilities for ovarian cancer mortality. Section D: Sensitivity analyses; definitions and results. Section E: Ovarian Cancer Searches and Results. (DOCX 66 kb)

## Abbreviations

EVPI: Expected value of perfect information
EVPPI: Expected value of perfect parameter information
HRQoL: Health-related quality of life
ICER: Incremental cost-effectiveness ratio
MMS: Multimodal screening
QALY: Quality-adjusted life year
ROCA: Risk of ovarian cancer algorithm
R-P: Royston-Parmar
TVS: Transvaginal ultrasound scan
UKCTOCS: United Kingdom Collaborative Trial of Ovarian Cancer Screening
USS: Ultrasound screening
