# Erratum to: Cost-effectiveness of screening for ovarian cancer amongst postmenopausal women: a model-based economic evaluation

**DOI:** 10.1186/s12916-017-0803-y

**Published:** 2017-02-03

**Authors:** Ben Kearns, Jim Chilcott, Sophie Whyte, Louise Preston, Susi Sadler

**Affiliations:** 1 The University of Sheffield, Regent Court, 30 Regent Street, Sheffield, S1 4DA UK; 2 University of Exeter Medical School, St Luke’s Campus, Heavitree Road, Exeter, EX1 2LU UK

## Erratum

After publication of the original article [1], it came to the authors’ attention that the Acknowledgements section was not completed correctly. Instead of “Not applicable”, the Acknowledgements of the article should have been as follows.

**Acknowledgements**

We would like to thank all of the following contributors for their invaluable expertise:

Neill Calvert for performing and writing-up the first draft of the literature reviews; Anne Mackie, John Marshall and members of the UK National Screening Committee; Usha Menon, Ian Jacobs, Andy Ryan and Alistair McGuire from the UKCTOCS; Jason Poole, Rebecca Elleray and Matthew Barclay from the Public Health England National Cancer Registration and Analysis Service; and Julia Palmer, Shahram Abdi, John Tidy and Simon Pledge from Sheffield Teaching Hospitals NHS Foundation Trust.

The views expressed in this publication are those of the authors and not necessarily those of any of the individuals or organisations named above.

## References

[1] Kearns B, Chilcott J, Whyte S, Preston L, Sadler S (2016). Cost-effectiveness of screening for ovarian cancer amongst postmenopausal women: a model-based economic evaluation. BMC Med.

