# Supplementary material for: Cost-effectiveness of screening for ovarian cancer amongst postmenopausal women: a model-based economic evaluation
Source: BMC Med. 2016 Dec 6;14:200. doi: 10.1186/s12916-016-0743-y (PMC5139096; doi:10.1186/s12916-016-0743-y)
Supplement: Additional file 1: — Section A: Derivation of utility values for the health economic model. Section B: Estimates of resource use and costs for the health economic model. Section C: Derivation of annual transition probabilities for ovarian cancer mortality. Section D: Sensitivity analyses; definitions and results. Section E: Ovarian Cancer Searches and Results. (DOCX 66 kb) [file 12916_2016_743_MOESM1_ESM.docx]

**Cost-effectiveness of screening for ovarian cancer amongst postmenopausal women; a model-based economic evaluation: Web extra material.**

**A Derivation of utility values for the health economic model.**

To derive the utility values used in the health economic model, the mean time trade-off values reported by Havrilesky *et al*^1^ were used, along with the following assumptions/steps:

1. The value reported for blood tests (0.90) represents the utility of women without ovarian cancer.
2. The value for early ovarian cancer (0.81) represents the utility of women diagnosed with stage 1 ovarian cancer who do not receive chemotherapy, but may or may not receive surgery.
3. The value for advanced ovarian cancer (0.55) represents the utility of women diagnosed with stage 4 ovarian cancer who do not receive chemotherapy, but may or may not receive surgery.
4. The values for women diagnosed with stages 2 or 3 ovarian cancer can be linearly interpolated from the values for stages 1 and 4. This gives values of (0.81 - 1/3 * [0.81 – 0.55] =) 0.723 for stage 2 and (0.81 - 2/3 * [0.81 – 0.55] =) 0.6373 for stage 3.
5. The impact of chemotherapy on HRQoL is as an additive disutility, and may be estimated as the difference between the utility for early ovarian cancer (0.81) and newly diagnosed ovarian cancer – chemotherapy grades 1 to 2 toxicity (0.60), giving a disutility of 0.21

To derive stage-specific treatment utilities, the values estimated at steps 1 to 4 were down-weighted by the chemotherapy disutility multiplied by the proportion of women who receive chemotherapy (based on data supplied by the English Cancer Registries^2^, as displayed in Table A1).

**Table A1 Utility values used in the economic model for women with ovarian cancer undergoing treatment.**

| Stage at diagnosis | Utility, no chemotherapy | Percent receiving chemotherapy | Utility |
| --- | --- | --- | --- |
| Stage I | 0.810 | 53% | 0.700 |
| Stage II | 0.723 | 71% | 0.575 |
| Stage III | 0.637 | 71% | 0.487 |
| Stage IV | 0.550 | 50% | 0.445 |
| Disutility to chemotherapy: | | 0.210 |  |

**B Estimates of resource use and costs for the health economic model.**

Table A2 provides estimates of resource use and cost for women diagnosed with ovarian cancer, or receiving a false positive diagnosis.

**Table A2 Resource components, use and cost amongst women receiving treatment for ovarian cancer, by stage at diagnosis.**

| **Diagnosis: resource components** | **Cost** | **Stage I** | **Stage II** | **Stage III** | **Stage IV** | **Borderline / No ovarian cancer** |
| --- | --- | --- | --- | --- | --- | --- |
| **Sheffield clinicians** |  |  |  |  |  |  |
| **Surgery** |  |  |  |  |  |  |
| Pelvic and peritoneal washings and biopsies | £1,909 | 5% | 5% | 5% | 5% | 5% |
| Total hysterectomy | £2,861 | 95% | 95% | 95% | 95% | 10% |
| Bilateral salpingo-oophorectomy | £1,418 | 100% | 100% | 95% | 95% | 100% |
| Infracolic-omentectomy | £1,909 | 100% | 100% | 100% | 100% | 100% |
| Pelvic and para-aortic lymph node sampling | £2,059 | 5% | 5% | 5% | 5% | 2% |
| Ultra-radical (extensive) surgery | £4,100 | 0% | 0% | 2% | 2% | 0% |
| Retroperitoneal lymph node dissection | £3,975 | 0% | 0% | 2% | 2% | 0% |
| **Total cost:** |  | **£6,243** | **£6,243** | **£6,334** | **£6,334** | **£3,750** |
| **Chemotherapy** |  |  |  |  |  |  |
| Carboplatin | £2,655 | 100% | 86% | 61% | 63% | N/A |
| Paclitaxel with carboplatin | £6,600 | 0% | 14% | 28% | 21% | N/A |
| Bevacizumab | £36,078 | 0% | 0% | 11% | 16% | N/A |
| **Total cost:** |  | **£2,655** | **£3,219** | **£7,465** | **£8,763** | **£0** |
|  |  |  |  |  |  |  |
| **Birmingham clinicians** |  |  |  |  |  |  |
| **Surgery** |  |  |  |  |  |  |
| Pelvic and peritoneal washings and biopsies | £1,909 | 5% | 5% | 5% | 5% | 5% |
| Total hysterectomy | £2,861 | 95% | 95% | 95% | 95% | 10% |
| Bilateral salpingo-oophorectomy | £1,418 | 100% | 100% | 95% | 95% | 100% |
| Infracolic-omentectomy | £1,909 | 100% | 100% | 100% | 100% | 100% |
| Pelvic and para-aortic lymph node sampling | £2,059 | 5% | 5% | 5% | 5% | 2% |
| Ultra-radical (extensive) surgery | £4,100 | 0% | 0% | 2% | 2% | 0% |
| Retroperitoneal lymph node dissection | £3,975 | 0% | 0% | 2% | 2% | 0% |
| **Total cost:** |  | **£6,243** | **£6,243** | **£6,334** | **£6,334** | **£3,750** |
| **Chemotherapy** |  |  |  |  |  |  |
| Carboplatin | £2,655 | 100% | 50% | 19% | 19% | N/A |
| Paclitaxel with carboplatin | £6,600 | 0% | 50% | 76% | 76% | N/A |
| Bevacizumab | £36,078 | 0% | 0% | 5% | 5% | N/A |
| **Total cost:** |  | **£2,655** | **£4,628** | **£7,252** | **£7,252** | **£0** |

N/A: Not applicable.

It is anticipated that if screening for ovarian cancer is implemented in England, then the resource use, with regards to the type and quantity of screens required is likely to be similar to that observed for the UKCTOCS trial^3^.

Within the UKCTOCS, first-level transvaginal scans (TVSs) were performed by ‘type 1’ sonographers, whilst second-level TVS were performed by ‘type 2’ sonographers. As type 2 sonographers are more experienced than type 1, the cost of a second-level TVS is likely to be greater than that of a first-level TVS. This cost difference may be estimated by assuming that type 1 sonographers are employed at Agenda for Change Band 7, and type 2 sonographers at Band 8a (these bands were frequently observed in published job adverts for sonographers and superintendent sonographers). Comparing pay at mid-points of the bands^4^, type 2 sonographers cost 21% more.

The cost of gynaecological ultrasound may be obtained from 2012/13 NHS reference costs^5^ (2013/14 reference costs do not distinguish between different types of ultrasound), under the HRG code RA24Z “Ultrasound Scan, 20 minutes and over”. There were 65,102 gynaecological-related examinations, of which all but 3 were amongst outpatient appointments. Of the outpatient examinations, 98% were classified as ‘gynaecological’ with a mean value of £55 (£43 to £65), the remaining 2% were classified as ‘gynaecological oncology’ with a mean value of £73 (£59 to £77). For the economic evaluation, the cost of a type 1 TVS was assumed to be equal to £55, inflated to a 2013/14 price (using HCHS indices^6^) of £55.82. The cost of a type 2 TVS was assumed to be 21% greater than this, at £67.55.

The cost of a CA-125 blood test was taken from cost estimates developed for the Early Cancer Detection Consortium^7^, which resulted in a cost of £54.19.

Overall costs per completed screen and per partial screen (for those who drop-out mid screening) were required for the economic model. The resource use (total number of level 1 and level 2 scans, along with repeat scans) for this was based on data presented in Figure 2 and 3 of Menon *et al*^3^. For completed screens, resource use was adjusted to account for the reported levels of drop-out. For example, in Figure 2 of Menon *et al* it was reported that of the 50,078 original level 1 scans for MMS, 8.6% (4,315) required a repeat scan but only 8.2% (4,121) attended their scan and of these first repeat scans a further 24.5% (1,008/4,121) required a second repeat scan but only 23.8% (979/4,121) attended. The number of repeat scans after adjusting for drop-out were then: first repeat; 4,315 (8.6% x 50,078) and second repeat 1,055 (24.5% x 4,315). Based on these calculations, the average number of level 1, level 2 and repeat scans required per completed screening episode for both types of screening are presented in Table A3.

**Table A3 Average number of level 1, level 2 and repeat screens per completed screening episode.**

| **Multimodal screening** | **Observed values** | | **Values with no drop-out** | |  |
| --- | --- | --- | --- | --- | --- |
|  | **Numbers screened** | **% who require another screen** | **Numbers screened** | **% of cohort** | **Screening cost** |
| Level 1 CA-125 screen | 50,078 | 8.6%  (4,315/50,078) | 50,078 | 100% | £54.19 |
| Level 1 - first repeat | 4,121 | 24.5%  (1,008/41,21) | 4,315  (50,078*8.6%) | 8.6% | £54.19 |
| Level 1 - second repeat | 979 | 9.8%  (96/979) | 1,055  (4,315*24.5%) | 2.1% | £54.19 |
| Level 2 screen | 409 | 38.1%  (156/409) | 430  (1,055*9.8%) | 0.9% | £67.55 |
| Level 2 - repeat | 132 | - | 164  (430*38.1%) | 0.3% | £67.55 |
|  |  |  |  | **Total cost:** | **£60.80** |
| **Ultrasound screening** | **Observed values** | | **Values with no drop-out** | |  |
|  | **Numbers screened** | **% who require another screen** | **Numbers screened** | **% of cohort** | **Screening cost** |
| Level 1 scan | 48,230 | 6.2%  (3,005/48,230) | 48,230 | 100% | £55.82 |
| Level 1 - first repeat | 2,779 | 4.1%  (115/2,779) | 3,005  (48,230*6.2%) | 6.2% | £55.82 |
| Level 2 screen | 2,785 | 1.6%  (45/2785) | 2,898  (4,315*4.1%) | 6.0% | £67.55 |
| Level 2 - first repeat | 42 | - | 47  (1,055*1.6%) | 0.1% | £67.55 |
|  |  |  |  | **Total cost:** | **£63.43** |

Data on observed values come from Figure 2 (multimodal screening) and Figure 3 (ultrasound screening) of Menon *et al*^3^.

The majority of drop-outs (205/266 for MMS and 328/356 for USS) occurred after the initial level-one screen. Therefore only the cost of the initial screen was used for drop-outs, giving an average cost of £54.19 for MMS and £55.82 for USS.

Estimates of the cost of end of life care were taken from Guest et al^8^, inflated to 2013/14 prices^6^.

For the probabilistic sensitivity analysis, estimates of uncertainty were required. For the costs of treatment, NHS reference costs^5^ for the procedure MA26 “Complex, Open or Laparoscopic, Upper or Lower Genital Tract Procedures for Malignancy” give a mean of £5,386 and an estimated standard error of £1,172. This standard error was used for all treatment costs. For diagnosis and end of life care, a standard error of 10% of the mean was assumed. Uncertainty in the cost of an ultrasound was taken from NHS reference costs^5^. Uncertainty in the cost of CA-125 was modelled by assuming an arbitrarily sample size of five and a range of £25 to £75, and using a beta distribution. All other costs were modelled using a Gamma distribution. For screening, level 1 and level 2 scans were separately sampled in the PSA. Hence the cost for those who complete screening is a hybrid combination of these two costs. Uncertainties in utilities and disutilities was modelled by assuming a sample size of 14 (the smallest reported sample from Havrilesky *et al*^1^), and using a beta distribution. Uncertainty in the number of false positives was modelled using data from UKCTOCS^9^ and beta distributions.

**C Derivation of annual transition probabilities for ovarian cancer mortality.**

Two main methods were used for deriving annual transition probabilities for ovarian cancer mortality. These were the use of exponential smoothing time-series methods (for extrapolating the hazard for no screening, and hazard ratios for both screening arms), and the use of standard parametric survival models. These are continuous functions, details on the derivation of annual transition probabilities are discussed for each method in turn.

*Time-series methods*.

Royston-Parmar models were fit to the digitized patient-level data using the ‘stmp2’ function in STATA version 14.1^10,11^. Five degrees of freedom were used for both the baseline hazard function and the time-dependent effect. Two models were fit: one to multimodal screening (MMS) and no screening, and one to ultrasound screening (USS) and no screening. From each model quarterly estimates of the hazard (for no screening) and hazard ratio (for active screening) were obtained. These were treated as a time-series, which were converted into an exponential smoothing state space model using the ‘ets’ function of the ‘forecast’ package in R^12^. Quarterly forecasts were obtained using the ‘forecast’ function (from the same package). Annual values, for use in the economic model, were obtained by taking the first quarter estimates for each year. Hazards were used directly as transition probabilities; hazard ratios for MMS and USS were derived by multiplying the hazard for no screening by the hazard ratio for screening.

*Standard parametric models.*

There were to approaches to using standard parametric models: either the same model structure was used for all three trial arms, or a separate model structure was considered for each arm. The best-fitting parametric models for MMS and USS was always the log-Normal. For no screening, the log-Normal was selected when fitting the same model structure to all arms, and the Weibull was selected when allowing model structure to vary with trial arm. These models were fit to the digitized patient-level data using the ‘streg’ function in STATA version 14.1^11^, reporting hazard ratios. Annual estimates of cumulative survival (CS) were estimated in Excel using the following formulae:

Weibull: “=EXP(‘_cons’*(*t*^EXP(‘/ln_p’)))”,

Log-Normal: =1-NORM.DIST(LN(*t*), ‘_cons’,EXP(‘/ln_sig’),TRUE)

Where ‘_cons’, ‘/ln_p’ and ‘/ln_sig’ are reported by STATA and *t* is the year.

The transition probability for year ‘x’ was calculated as: (CS[x-1]-CS[x])/ CS[x-1]

**D Sensitivity analyses; definitions and results.**

The following sensitivity analyses were performed:

- No screening results in the same false-positive rate as MMS

In the base-case analysis no screening is not associated with any false positives.

- Treatment costs monotonically increase with stage at diagnosis.

Costs were changed to always monotonically increase, by 10% of the previous stage’s cost (the costs for Stage I were the same as the base-case.

- Use Birmingham diagnosis and treatment cost estimates.

These are alternative cost estimates to the Sheffield estimates used in the base-case.

- No end of life costs

The cost associated with a death from ovarian cancer was removed.

- Alternative estimates of MMS

Two sensitivity analyses considered alternative ‘high’ and ‘low’ costs of £67.33 and £31.19.

- Include an end-of-life disutility for women who die from ovarian cancer.

A disutility of 0.4 for one year was modelled.

- Earlier treatment leads to reduced utility.

The effect of a disutility of 0.2 for the first year following diagnosis was evaluated.

- Include a disutility of 0.01 per year for people receiving a screen.

This is equivalent to experiencing a disutility of 0.52 for a week, or 0.12 for a month.

- Include a disutility of 0.005 per year for people receiving a screen.

This is equivalent to experiencing a disutility of 0.26 for a week, or 0.06 for a month.

- Include a cost for the ROCA Test of £150 per use.

This is the cost stated at: *https://www.therocatest.co.uk/roca-test/patient-faqs/’, question: ‘Ordering the ROCA Test: 1. How much does the ROCA Test cost? (last accessed 11^th^ October 2016)*.

Results of the sensitivity analyses are presented in Table A4.

**Table A4 Results from deterministic one-way sensitivity analyses.**

| **Base-case results** | **No screening** | **MMS** | **USS** |
| --- | --- | --- | --- |
| QALYs | 14.281 | 14.330 | 14.291 |
| Costs | £176 | £593 | £811 |
| ICER | - | £8,459 vs no screening | Dominated by MMS |
| **False-positives for no screening same as for MMS** | **No screening** | **MMS** | **USS** |
| QALYs | 14.277 | 14.330 | 14.291 |
| Costs | £237 | £593 | £811 |
| ICER |  | £6,691 vs no screening | Dominated by MMS |
| **Treatment costs monotonically increase by 10%** | **No screening** | **MMS** | **USS** |
| QALYs | 14.281 | 14.330 | 14.291 |
| Costs | £179 | £594 | £814 |
| ICER |  | £8,423 vs no screening | Dominated by MMS |
| **Birmingham treatment cost estimates** | **No screening** | **MMS** | **USS** |
| QALYs | 14.281 | 14.330 | 14.291 |
| Costs | £180 | £596 | £815 |
| ICER |  | £8,454 vs no screening | Dominated by MMS |
| **No end of life costs** | **No screening** | **MMS** | **USS** |
| QALYs | 14.281 | 14.330 | 14.291 |
| Costs | £69 | £546 | £735 |
| ICER |  | £9,670 vs no screening | Dominated by MMS |
| **Low-cost estimate for MMS** | **No screening** | **MMS** | **USS** |
| QALYs | 14.281 | 14.330 | 14.291 |
| Costs | £176 | £426 | £811 |
| ICER |  | £5,071 vs no screening | Dominated by MMS |
| **High-cost estimate for MMS** | **No screening** | **MMS** | **USS** |
| QALYs | 14.281 | 14.330 | 14.291 |
| Costs | £176 | £688 | £811 |
| ICER |  | £10,394 vs no screening | Dominated by MMS |
| **End-of-life disutility for ovarian cancer deaths** | **No screening** | **MMS** | **USS** |
| QALYs | 14.275 | 14.327 | 14.286 |
| Costs | £176 | £593 | £811 |
| ICER | - | £8,020 vs no screening | Dominated by MMS |
| **Earlier treatment leads to reduced utility** | **No screening** | **MMS** | **USS** |
| QALYs | 14.281 | 14.328 | 14.289 |
| Costs | £176 | £593 | £811 |
| ICER | - | £8,773 vs no screening | Dominated by MMS |
| **Annual screening disutility of 0.01** | **No screening** | **MMS** | **USS** |
| QALYs | 14.281 | 14.263 | 14.227 |
| Costs | £176 | £593 | £811 |
| ICER | - | Dominated by no screening | Dominated by no screening |
| **Annual screening disutility of 0.005** | **No screening** | **MMS** | **USS** |
| QALYs | 14.281 | 14.297 | 14.259 |
| Costs | £176 | £593 | £811 |
| ICER | - | £26,173 vs no screening | Dominated by MMS |
| **ROCA Test cost £150 per use** | **No screening** | **MMS** | **USS** |
| QALYs | 14.281 | 14.330 | 14.291 |
| Costs | £176 | £1,682 | £811 |
| ICER | - | £30,552 vs no screening | Extendedly dominated by MMS |

Ovarian Cancer Searches and Results

All searches were performed during September and October 2014.

The search results by database are provided in Table A5.

**Table A5 Search results by database.**

| **Database** | **RCTs($$rct)** | **Economic and Quality of Life ($$economicqol)** |
| --- | --- | --- |
| Medline | 1114 | 324 and 345 = 669 (642 deduplicated) |
| Embase | 3054 | 558 (Quality of Life)  890 (Economics)= 1448 (1400 deduplicated) |
| CINAHL | 238 | 313 and 160 = 473 (455 deduplicated) |
| Web of Science | 490 | 473 (183 records after deduplication) |
| Cochrane CENTRAL | 195 | Records come from Medline so not searched. |
| Cochrane NHS EED | Not searched | 80 |
| Cochrane CDSR | 6 | Not searched |
| Cochrane DARE | 5 | Not searched |
| Cochrane HTA | 15 | Not searched |
| Cochrane Methods | 8 | Not searched |
| Econlit | Not searched | 11 |
| Total | 5125 | 2771 |
| Total after deduplication* | 2716 | 2089 |

**Search One - Economic and Quality of Life ($$economicqol)**

Reade Ovarian Cancer terms AND Quality of Life terms.

OR

Reade Ovarian Cancer terms AND screening terms AND economic filter.

**Search Two - RCTs($$rct)**

Reade Ovarian Cancer terms AND Reade screening terms (n.b we will be excluding the terms from the Reade search relating to hereditary cancers).

2011-2014.

No filter.

Limit to Humans and English Language

*Deduplication also involved removal of pre 2011 papers from the RCT search, removal of obvious non English studies and removal of obvious animal studies. However these were not searched for systematically so errors may remain.

Reade Ovarian Cancer Terms (include tubal and peritoneal terms but exclude hereditary terms)

1. Exp Ovarian Neoplasms/

2. Fallopian Tube Neoplasms/

3. Peritoneal Neoplasms/

4. (fallopian tube adj (neoplasm* or cancer* or tumo* or carcinoma*)).mp

5. (peritoneal adj (neoplasm* or cancer* or tumo* or carcinoma*)).mp

6. (ovarian adj3 cancer*).mp

7. (ovarian adj3 neoplas*).mp

8. (ovarian adj3 tumo*).mp

9. (ovarian adj3 carcinoma*).mp

10. (ovarian adj3 adenocarcinoma*).mp

Screening Terms

1 Tumor Markers, Biological/

2. Biological Markers/

3. CA-125 Antigen/

4. Mass Screening/

5. (screen$ or test$ or imag$ or predict$ or surveillance).tw.

6. "Early Detection of Cancer"/

7. (earl$ adj (diagnos$ or detect$)).ti,ab.

Reade screening terms

1. Mass Screening/

2. "Early Detection of Cancer"/

3. screen*.mp

RCT filter

1. Randomized controlled trials as Topic/

2. Randomized controlled trial/

3. Random allocation/

4. randomized controlled trial.pt.

5. Double blind method/

6. Single blind method/

7. Clinical trial/

8. exp Clinical Trials as Topic/

9. controlled clinical trial.pt.

10. clinical trial$.pt.

11. multicenter study.pt.

12. or/1-12

13. (clinic$ adj25 trial$).ti,ab.

14. ((singl$ or doubl$ or treb$ or tripl$) adj (blind$ or mask$)).tw.

15. Placebos/

16. Placebo$.tw.

17. (allocated adj2 random).tw.

18. or/13-18

19. 12 or 18

20. Case report.tw.

21. Letter/

22. Historical article/

23. 20 or 21 or 22

24. exp Animals/

25. Humans/

26. 24 not (24 and 25)

27. 23 or 26

28. 19 not 27

Economic evaluation filter

1. exp "Costs and Cost Analysis"/

2. Economics/

3. exp Economics, Hospital/

4. exp Economics, Medical/

5. Economics, Nursing/

6. exp models, economic/

7. Economics, Pharmaceutical/

8. exp "Fees and Charges"/

9. exp Budgets/

10. budget$.tw.

11. ec.fs.

12. cost$.ti.

13. (cost$ adj2 (effective$ or utilit$ or benefit$ or minimi$)).ab.

14. (economic$ or pharmacoeconomic$ or pharmaco-economic$).ti.

15. (price$ or pricing$).tw.

16. (financial or finance or finances or financed).tw.

17. (fee or fees).tw.

18. (value adj2 (money or monetary)).tw.

19. quality-adjusted life years/

20. (qaly or qalys).af.

21. (quality adjusted life year or quality adjusted life years).af.

22 or/1-21

Quality of Life terms

1.(EORTC or QLQ-C30).mp.

2.FACT or functional assessment of cancer therapy or FLIC or (FACT adj3 cancer)

3. RSCL or SDS or Fact-G or CNQ-SF or CNQ or CARES-SF or CARES or ESAS

4.quality adjusted life year/ or quality adjusted life.tw. or (qaly$ or qald$ or qale$ or qtime$).tw.

4. disability adjusted life.tw. or daly$.tw.

5. health status indicators/

6. (sf36 or sf 36 or short form 36 or shortform 36 or sf thirtysix or sf thirty six or shorform thirtysix or shortform thirty six or short form thirtysix or short form thirty six).tw.

7. (sf 6 or sf6 or short form 6 or shortform 6 or sf six or sfsix or shortform six or short form six).tw.

8. (sf12 or sf 12 or short form 12 or shortform 12 or sf twelve or sftwelve or shortform twelve or short form twelve).tw.

9. (sf16 or sf 16 or short form 16 or shortform 16 or sf sixteen or sfsixteen or shortfrom sixteen or short form sixteen).tw.

10. (sf20 or sf 20 or short form 20 or shortform 20 or sf twenty or sftwenty or shortform twenty or short form twenty).tw.

11. (euroqol or euro qol or eq5d or eq 5d).tw.

12. (hql or hqol or h qol or hrqol or hr qol).tw.

13. (hye or hyes).tw. or ( health$ year$ equivalent$).tw.

14. health utilit$.tw. or (hui or hui1 or hui2 or hui3).tw.

15. ("Quality of Life" or “Outcome Assessment (Health Care)"/ or quality of life.tw.) AND (preference based or utilit$ or generic preference).tw.

Ovid MEDLINE(R) In-Process & Other Non-Indexed Citations and Ovid MEDLINE(R) 1946 to Present

1. exp Ovarian Neoplasms/

2. Fallopian Tube Neoplasms/

3. Peritoneal Neoplasms/

4. (fallopian tube* adj (neoplasm* or cancer* or tumo* or carcinoma*)).mp.

5. (peritoneal adj (neoplasm* or cancer* or tumo* or carcinoma*)).mp.

6. ovarian cancer*.mp.

7. ovarian tumo*.mp.

8. ovarian carcinoma*.mp.

9. ovarian adenocarcinoma*.mp.

10. ovarian neoplas*.mp.

11. 1 or 2 or 3 or 4 or 5 or 6 or 7 or 8 or 9 or 10

12. (EORTC or QLQ-C30).mp.

13. ((FACT or FLIC) adj3 cancer).mp.

14. ((RSCL or SDS or Fact-G or CNQ-SF or CNQ or CARES-SF or CARES or ESAS) adj3 cancer*).mp.

15. quality adjusted life year/ or quality adjusted life.mp. or (qaly* or qald* or qale* or qtime*).mp.

16. (disability adjusted life or daly$).mp.

17. health status indicators/

18. (sf36 or sf 36 or short form 36 or shortform 36 or sf thirtysix or sf thirty six or shorform thirtysix or shortform thirty six or short form thirtysix or short form thirty six).mp.

19. (sf 6 or sf6 or short form 6 or shortform 6 or sf six or sfsix or shortform six or short form six).mp.

20. (sf12 or sf 12 or short form 12 or shortform 12 or sf twelve or sftwelve or shortform twelve or short form twelve).mp.

21. (sf16 or sf 16 or short form 16 or shortform 16 or sf sixteen or sfsixteen or shortfrom sixteen or short form sixteen).mp.

22. (sf20 or sf 20 or short form 20 or shortform 20 or sf twenty or sftwenty or shortform twenty or short form twenty).mp.

23. (euroqol or euro qol or eq5d or eq 5d).mp.

24. (hql or hqol or h qol or hrqol or hr qol).mp.

25. (hye or hyes or health* year* equivalent*).mp.

26. (health utilit* or (hui or hui1 or hui2 or hui3)).mp.

27. (Quality of Life/ or Outcome Assessment/) and (preference based or utilit* or generic preference).mp.

28. 12 or 13 or 14 or 15 or 16 or 17 or 18 or 19 or 20 or 21 or 22 or 23 or 24 or 25 or 26 or 27

29. 11 and 28

30. limit 29 to (english language and humans)

31. Tumor Markers, Biological/

32. Biological Markers/

33. CA-125 Antigen/

34. Mass Screening/

35. "Early Detection of Cancer"/

36. (screen$ or test$ or imag$ or predict$ or surveillance).tw.

37. (earl$ adj (diagnos$ or detect$)).ti,ab.

38. Early Detection of Cancer/

39. 31 or 32 or 33 or 34 or 35 or 36 or 37 or 38

40. exp "Costs and Cost Analysis"/

41. exp Economics, Hospital/

42. exp Economics, Medical/

43. Economics, Nursing/

44. exp models, economic/

45. Economics, Pharmaceutical/

46. exp "Fees and Charges"/

47. exp Budgets/

48. budget$.tw.

49. ec.fs.

50. cost$.ti.

51. (cost$ adj2 (effective$ or utilit$ or benefit$ or minimi$)).ab.

52. (economic$ or pharmacoeconomic$ or pharmaco-economic$).ti.

53. (price$ or pricing$).tw.

54. (financial or finance or finances or financed).tw.

55. (fee or fees).tw.

56. (value adj2 (money or monetary)).tw.

57. quality-adjusted life years/

58. (qaly or qalys).af.

59. (quality adjusted life year or quality adjusted life years).af.

60. 40 or 41 or 42 or 43 or 44 or 45 or 46 or 47 or 48 or 49 or 50 or 51 or 52 or 53 or 54 or 55 or 56 or 57 or 58 or 59

61. 11 and 39 and 60

62. limit 61 to (english language and humans)

63. 30 or 62

Embase 1988

1. exp Ovarian Neoplasms/

2. Fallopian Tube Neoplasms/

3. Peritoneal Neoplasms/

4. (fallopian tube* adj (neoplasm* or cancer* or tumo* or carcinoma*)).mp.

5. (peritoneal adj (neoplasm* or cancer* or tumo* or carcinoma*)).mp.

6. ovarian cancer*.mp.

7. ovarian tumo*.mp.

8. ovarian carcinoma*.mp.

9. ovarian adenocarcinoma*.mp.

10. ovarian neoplas*.mp.

11. 1 or 2 or 3 or 4 or 5 or 6 or 7 or 8 or 9 or 10

12. (EORTC or QLQ-C30).mp.

13. ((FACT or FLIC) adj3 cancer).mp.

14. ((RSCL or SDS or Fact-G or CNQ-SF or CNQ or CARES-SF or CARES or ESAS) adj3 cancer*).mp.

15. quality adjusted life year/ or quality adjusted life.mp. or (qaly* or qald* or qale* or qtime*).mp.

16. (disability adjusted life or daly$).mp.

17. health status indicators/

18. (sf36 or sf 36 or short form 36 or shortform 36 or sf thirtysix or sf thirty six or shorform thirtysix or shortform thirty six or short form thirtysix or short form thirty six).mp.

19. (sf 6 or sf6 or short form 6 or shortform 6 or sf six or sfsix or shortform six or short form six).mp.

20. (sf12 or sf 12 or short form 12 or shortform 12 or sf twelve or sftwelve or shortform twelve or short form twelve).mp.

21. (sf16 or sf 16 or short form 16 or shortform 16 or sf sixteen or sfsixteen or shortfrom sixteen or short form sixteen).mp.

22. (sf20 or sf 20 or short form 20 or shortform 20 or sf twenty or sftwenty or shortform twenty or short form twenty).mp.

23. (euroqol or euro qol or eq5d or eq 5d).mp.

24. (hql or hqol or h qol or hrqol or hr qol).mp.

25. (hye or hyes or health* year* equivalent*).mp.

26. (health utilit* or (hui or hui1 or hui2 or hui3)).mp.

27. (Quality of Life/ or Outcome Assessment/) and (preference based or utilit* or generic preference).mp.

28. 12 or 13 or 14 or 15 or 16 or 17 or 18 or 19 or 20 or 21 or 22 or 23 or 24 or 25 or 26 or 27

29. 11 and 28

30. limit 29 to (english language and humans)

31. Tumor Markers, Biological/

32. Biological Markers/

33. CA-125 Antigen/

34. Mass Screening/

35. "Early Detection of Cancer"/

36. (screen$ or tested or testing or test or tests or imag$ or predict$ or surveillance).tw.

37. (earl$ adj (diagnos$ or detect$)).ti,ab.

38. Early Detection of Cancer/

39. 31 or 32 or 33 or 34 or 35 or 36 or 37 or 38

40. exp "Costs and Cost Analysis"/

41. exp Economics, Hospital/

42. exp Economics, Medical/

43. Economics, Nursing/

44. exp models, economic/

45. Economics, Pharmaceutical/

46. exp "Fees and Charges"/

47. exp Budgets/

48. 40 or 41 or 42 or 43 or 44 or 45 or 46 or 47

49. 11 and 39 and 48

50. limit 49 to (human and english language)

51. 30 or 50

Econlit

Limited search to Ovarian Cancer terms only. The only ovarian cancer term that had any hits was ovarian cancer*.mp. so the search was limited to this.

1. Ovarian cancer.mp

CINAHL

S48 S28 OR S47

S47 S37 AND S46

S46 (S38 OR S39 OR S40 OR S41 OR S42 OR S43 OR S44 OR S45)

S45 TX (value N2 (money or monetary))

S44 TX fee or fees

S43 TX financial or finance or finances or financed

S42 TX price? or pricing?

S41 TX economic* or pharmacoeconomic* or pharmaco-economic*

S40 TX cost*

S39 TX budget*

S38 MH economics

S37 S4 AND S36

S36 S29 OR S30 OR S31 OR S32 OR S33 OR S34 OR S35

S35 TX earl* N (diagnos* or detect*)

S34 TX screen* or test* or imag* or predict* or surveillance

S33 MH Early Detection of Cancer

S32 MH Mass Screening

S31 MH CA-125 Antigen

S30 MH Biological Markers

S29 MH Tumor Markers, Biological

S28 (S4 AND S27)

S27 S5 OR S6 OR S7 OR S8 OR S9 OR S10 OR S11 OR S12 OR S13 OR S14 OR S15 OR S16 OR S17 OR S18 OR S19 OR S20 OR S21 OR S22 OR S23 OR S24 OR S26

S26 (TX ( preference based or utilit* or generic preference )) AND (S24 AND S25)

S25 TX preference based or utilit* or generic preference

S24 MH Outcome Assessment

S23 MH Quality of Life

S22 (health utilit* or (hui or hui1 or hui2 or hui3))

S21 hye or hyes or health* year* equivalent*

S20 TX hql or hqol or h qol or hrqol or hr qol

S19 TX euroqol or euro qol or eq5d or eq 5d

S18 TX sf20 or sf 20 or short form 20 or shortform 20 or sf twenty or sftwenty or shortform twenty or short form twenty

S17 TX sf16 or sf 16 or short form 16 or shortform 16 or sf sixteen or sfsixteen or shortfrom sixteen or short form sixteen

S16 TX sf12 or sf 12 or short form 12 or shortform 12 or sf twelve or sftwelve or shortform twelve or short form twelve

S15 TX sf 6 or sf6 or short form 6 or shortform 6 or sf six or sfsix or shortform six or short form six

S14 TX (sf36 or sf 36 or short form 36 or shortform 36 or sf thirtysix or sf thirty six or shorform thirtysix or shortform thirty six or short form thirtysix or short form thirty six)

S13 MH health status indicators

S12 TX disability adjusted life or daly*

S11 TX qaly* or qald* or qale* or qtime*

S10 TX quality adjusted life

S9 MH qaly

S8 MH quality adjusted life year

S7 TX ((RSCL or SDS or Fact-G or CNQ-SF or CNQ or CARES-SF or CARES or ESAS) N3 cancer*)

S6 TX ((FACT or FLIC) N3 cancer)

S5 TX (EORTC or QLQ-C30)

S4 S1 OR S2 OR S3

S3 TX ((fallopian tube* or peritoneal or ovarian) N1 (neoplasm* or cancer* or tumo* or carcinoma* or adenocarcinoma))

S2 MH Peritoneal Neoplasms

S1 MH ovarian neoplasms

Cochrane Library

#1 MeSH descriptor: [Ovarian Neoplasms] explode all trees

#2 MeSH descriptor: [Peritoneal Neoplasms] explode all trees

#3 MeSH descriptor: [Fallopian Tube Neoplasms] explode all trees

#4 ((peritoneal or ovarian or fallopian) near/1 (neoplasm* or cancer* or tumo* or carcinoma* or adenocarcinoma*)):ti,ab,kw (Word variations have been searched)

#5 #1 or #2 or #3 or #4

#6 (qaly* or qald* or qale* or qtime* or daly):ti,ab,kw (Word variations have been searched)

#7 ((disability or quality) near (adjusted life year*)):ti,ab,kw

#8 sf36 or sf 36 or short form 36 or shortform 36 or sf thirtysix or sf thirty six or shorform thirtysix or shortform thirty six or short form thirtysix or short form thirty six or sf 6 or sf6 or short form 6 or shortform 6 or sf six or sfsix or shortform six or short form six or sf12 or sf 12 or short form 12 or shortform 12 or sf twelve or sftwelve or shortform twelve or short form twelve or sf16 or sf 16 or short form 16 or shortform 16 or sf sixteen or sfsixteen or shortfrom sixteen or short form sixteen or sf20 or sf 20 or short form 20 or shortform 20 or sf twenty or sftwenty or shortform twenty or short form twenty:ti,ab,kw

#9 euroqol or euro qol or eq5d or eq 5d or hql or hqol or h qol or hrqol or hr qol or hye or hyes or health* year* equivalent* or health utilit* or hui or hui1 or hui2 or hui3:ti,ab,kw

#10 MeSH descriptor: [Quality-Adjusted Life Years] explode all trees

#11 #5 or #6 or #7 or #8 or #9 or #10

#12 #5 and #11

#13 MeSH descriptor: [Tumor Markers, Biological] explode all trees

#14 MeSH descriptor: [Biological Markers] explode all trees

#15 MeSH descriptor: [Mass Screening] explode all trees

#16 MeSH descriptor: [Early Detection of Cancer] explode all trees

#17 screen$ or test$ or imag$ or predict$ or surveillance:ti,ab,kw

#18 (earl* near (diagnos* or detect*)):ti,ab,kw

#19 #13 or #14 or #15 or #16 or #17 or #18

#20 #5 and #19

#21 #12 or #20

Web of Science

# 8 #7 AND #6 AND #1

# 7 TITLE: (Cost* or economic* or budget* or price or pricing or financial or finance or finances or financed or fee or fees or money or monetary)

# 6 TITLE: (((screen$ or tested or testing or test or tests or imag$ or predict$ or surveillance) or (earl$ NEAR (diagnos$ or detect$))))

# 5 #4 AND #1

# 4 #3 OR #2

# 3 TITLE: ((qaly* or qald* or qale* or qtime or daly$ or sf36 or sf 36 or short form 36 or shortform 36 or sf thirtysix or sf thirty six or shorform thirtysix or shortform thirty six or short form thirtysix or short form thirty six or sf 6 or sf6 or short form 6 or shortform 6 or sf six or sfsix or shortform six or short form six or sf12 or sf 12 or short form 12 or shortform 12 or sf twelve or sftwelve or shortform twelve or short form twelve or sf16 or sf 16 or short form 16 or shortform 16 or sf sixteen or sfsixteen or shortfrom sixteen or short form sixteen or sf20 or sf 20 or short form 20 or shortform 20 or sf twenty or sftwenty or shortform twenty or short form twenty or euroqol or euro qol or eq5d or eq 5d or hql or hqol or h qol or hrqol or hr qol or hye or hyes or (health* year* equivalent*) or health utilit* or hui or hui1 or hui2 or hui3))

# 2 TITLE: ((EORTC or QLQ-C30 or ((FACT or FLIC or RSCL or SDS or Fact-G or CNQ-SF or CNQ or CARES-SF or CARES or ESAS) NEAR/2 (cancer*))))

# 1 TITLE: ((peritoneal or ovarian or fallopian) NEAR/1 (neoplasm* or cancer* or tumo* or carcinoma* or adenocarcinoma*))

Medline and Embase RCTs

1. exp Ovarian Neoplasms/

2. Fallopian Tube Neoplasms/

3. Peritoneal Neoplasms/

4. (fallopian tube adj (neoplasm* or cancer* or tumo* or carcinoma*)).mp.

5. (peritoneal adj (neoplasm* or cancer* or tumo* or carcinoma*)).mp.

6. (ovarian adj3 cancer*).mp.

7. (ovarian adj3 neoplas*).mp.

8. (ovarian adj3 tumo*).mp.

9. (ovarian adj3 carcinoma*).mp.

10. (ovarian adj3 adenocarcinoma*).mp.

11. 1 or 2 or 3 or 4 or 5 or 6 or 7 or 8 or 9 or 10

12. Mass Screening/

13. "Early Detection of Cancer"/

14. screen*.mp.

15. 12 or 13 or 14

16. 11 and 15

17. limit 16 to yr="2011 -Current"

18. limit 17 to (human and english language)

CINAHL

S9 (S4 OR S5 OR S6) AND (S7 AND S8)

S8 S4 OR S5 OR S6

S7 S1 OR S2 OR S3

S6 TX screen*

S5 MJ detection

S4 MJ screening

S3 TX ((fallopian tube* or peritoneal or ovarian) N3 (neoplasm* or cancer* or tumo* or carcinoma* or adenocarcinoma))

S2 MH peritoneal neoplasms

S1 MH ovarian neoplasms

Web of Science

# 5 #2 AND #1

Refined by: PUBLICATION YEARS: (2013 OR 2011 OR 2012 OR 2014) AND DOCUMENT TYPES: (ARTICLE)

# 4 #2 AND #1

# 3 #2 AND #1

# 2 TI=(screen or screening or detect or detecting or detection or detects)

# 1 TS=((peritoneal or ovarian or fallopian) NEAR/1 (neoplasm* or cancer* or tumo* or carcinoma* or adenocarcinoma*))

Cochrane

#1 MeSH descriptor: [Ovarian Neoplasms] explode all trees

#2 MeSH descriptor: [Peritoneal Neoplasms] explode all trees

#3 MeSH descriptor: [Fallopian Tube Neoplasms] explode all trees

#4 ((peritoneal or ovarian or fallopian) near/1 (neoplasm* or cancer* or tumo* or carcinoma* or adenocarcinoma*)):ti,ab,kw (Word variations have been searched)

#5 MeSH descriptor: [Mass Screening] explode all trees

#6 MeSH descriptor: [Early Detection of Cancer] explode all trees

#7 screen*:ti,ab,kw

#8 (1 or 2 or 3 or 4) and (5 or 6 or 7)

**References**

1. Havrilesky L.J., Broadwater G., Davis D.M., Nolte K.C., Barnett J.C., Myers E.R. et al. Determination of quality of life-related utilities for health states relevant to ovarian cancer diagnosis and treatment. *Gynecologic oncology* 113.2 (2009): 216-220

2. Public Health England. Data from the English Cancer Registries (Personal Communication). 2015.

3. Menon U., Gentry-Maharaj A., Hallett R., Ryan A., Burnell M., Sharma A. et al. Sensitivity and specificity of multimodal and ultrasound screening for ovarian cancer, and stage distribution of detected cancers: results of the prevalence screen of the UK Collaborative Trial of Ovarian Cancer Screening (UKCTOCS). *The Lancet Oncology* 2009; 10(4):327-340.

4. NHS Careers. Agenda for change - pay rates. 2015.<http://www.nhscareers.nhs.uk/working-in-the-nhs/pay-and-benefits/agenda-for-change-pay-rates/> Accessed 11 October 2016.

5. Department of Health. NHS reference costs 2012 to 2013. 2015.https://[www.gov.uk/government/publications/nhs-reference-costs-2012-to-2013](http://www.gov.uk/government/publications/nhs-reference-costs-2012-to-2013) Accessed 11 October 2016.

6. Curtis L. PSSRU Unit Costs of Health & Social Care 2013. *University of Kent (United Kingdom)* 2013.

7. Whyte S, Pollard D, Cree I. **Generic Cancer Screen - Economic modelling report**. The University of Sheffield. Health Economics & Decision Science Discussion Paper Series. https://www.shef.ac.uk/scharr/sections/heds/discussion-papers/16_11-1.580331 Accessed 11 October 2016.

8. Guest J.F., Ruiz F.J., Greener M.J., Trotman I.F. Palliative care treatment patterns and associated costs of healthcare resource use for specific advanced cancer patients in the UK. *European Journal of Cancer Care* 2006; 15(1):65-73.

9. Jacobs I.J., Menon U., Ryan A., Gentry-Maharaj A., Burnell M., Kalsi J.K. et al. Ovarian cancer screening and mortality in the UK Collaborative Trial of Ovarian Cancer Screening (UKCTOCS): a randomised controlled trial. *The Lancet* 2015.

10. Royston P., Parmar M.K. Flexible parametric proportional-hazards and proportional-odds models for censored survival data, with application to prognostic modelling and estimation of treatment effects. *Statistics in Medicine* 2002; 21(15):2175-2197.

11. StataCorp. Stata Statistical Software: Release 14. 2015, College Station, TX: StataCorp LP.

12. Hyndman RJ, Khandakar Y. Automatic time series for forecasting: the forecast package for R. Journal of Statistical Software 2007, 27(3)
